# A randomized placebo-controlled phase II study of a *Pseudomonas* vaccine in ventilated ICU patients

**DOI:** 10.1186/s13054-017-1601-9

**Published:** 2017-02-04

**Authors:** Jordi Rello, Claus-Georg Krenn, Gottfried Locker, Ernst Pilger, Christian Madl, Laura Balica, Thierry Dugernier, Pierre-Francois Laterre, Herbert Spapen, Pieter Depuydt, Jean-Louis Vincent, Lajos Bogár, Zsuzsanna Szabó, Barbara Völgyes, Rafael Máñez, Nahit Cakar, Atilla Ramazanoglu, Arzu Topeli, Maria A. Mastruzzo, Abel Jasovich, Christian G. Remolif, Liliana del Carmen Soria, Max A. Andresen Hernandez, Carolina Ruiz Balart, Ildikó Krémer, Zsolt Molnár, Frank von Sonnenburg, Arthur Lyons, Michael Joannidis, Heinz Burgmann, Tobias Welte, Anton Klingler, Romana Hochreiter, Kerstin Westritschnig

**Affiliations:** 1 Hospital Universitari Joan XXIII, C. Dr. Mallafrè Guasch 4, 43007 Tarragona, Spain; 2 CIBERES, Hospital Universitari Vall d’Hebron, Passeig Vall d’Hebron, 119, 08035 Barcelona, Spain; 3 Medical University of Vienna, Intensive Care 13C1, Währinger Gürtel 18 – 20, 1090 Vienna, Austria; 4 Department of Internal Medicine I, Medical University of Vienna, Intensive Care 13I2, Währinger Gürtel 18 – 20, 1090 Vienna, Austria; 5 Intensive Care, Department of Internal Medicine, University Hospital Graz, Auenbruggerplatz 15, 8036 Graz, Austria; 6 Department of Internal Medicine III, Intensive Care 13H1, Medical University of Vienna, Währinger Gürtel 18 – 20, 1090 Vienna, Austria; 7 Emergency Clinical Hospital Bucharest, Toxicology – ICU, 8 Floreasca Street, 01446 Bucharest, Romania; 8 Clinique St. Pierre, Intensive Care Department, Avenue Reine Fabiola 9, 1340 Ottignies, Belgium; 9 Department of CCM, St. Luc University Hospital UCL, Université Catholique de Louvain, Avenue Hippocrate 10, 1200 Brussels, Belgium; 10 University Hospital Vrije Universiteit Brussels, Laarbeeklaan 101, 1090 Brussels, Belgium; 11 UZ Gent, De Pintelaan 185, 9000 Gent, Belgium; 12 Erasme University Hospital, Université Libre de Bruxelles, Route de Lennik 808, 1070 Brussels, Belgium; 13 University of Pécs Anesthesiology and Intensive Care Department, Ifjúság ut 13, 7624 Pécs, Hungary; 14 Uzsoki Hospital, Uzsoki u. 29, 1145 Budapest, Hungary; 15 Bajcsy Zsilinszky Hospital and Polyclinic, Intensive Care Unit, Maglodi út 89-91, 1106 Budapest, Hungary; 16 Department for Critical Care Medicine, Bellvitge University Hospital, Feixa Llarga s/n, 08907, L’Hospitalet de Llobregat, Barcelona, Spain; 17 Department of Anesthesiology and Reanimation, Istanbul University Capa Medical Faculty, 34390 Istanbul, Turkey; 18 Department of Anesthesiology, Dumlupinar Bulvari Kampus Antalya, Akdeniz University, Faculty of Medicine Hospital, 07070 Antalya, Turkey; 19 Department of Internal Medicine, Intensive Care Unit, Hacettepe University Hospital, 06100 Ankara, Turkey; 20 Hospital Dr. Carlos Bocalandro, Ruta 8 No. 9100, B1657BHD Loma Hermosa, Partido 3 de Febrero, Buenos Aires, Argentina; 21 Sanatorio Güemes, Av. Roque Sanchez Pena 811 5°C, C1035AAP Buenos Aires, Argentina; 22 Hospital “Heroes de Malvinas”, Av. Ricardo Balbín 1910, B1721FJN Merlo, Buenos Aires, Argentina; 23 Hospital Central Mendoza, Alem y Salta, M5500GKO Ciudad de Mendoza, Argentina; 24 Hospital Clinico, Facultad de Medicina Pontificia, Universidad Católica de Chile, Marcoleta 367, Santiago, Chile; 25 Hospital Dr. Sótero del Rio, Unidad de Cuidado Intensivo, Departamento de Medicina Intensiva, Escuela de Medicina, Pontificia Universidad Católica de Chile, Avenida Concha y Toro, 3459 Puente Alto, Santiago, Chile; 26 Flor Ferenc County Hospital, Semmelweis tér 1, 2143 Kistarcsa, Hungary; 27 Department of Anaesthesia and Intensive Care, University of Szeged, Semmelweis u. 6, 6720 Szeged, Hungary; 28 Department of Infectious Diseases and Tropical Medicine, University of Munich, Georgenstr. 5, 80799 Munich, Germany; 29 Clinical Research Department, Division of Virus Diseases, Walter Reed Army Institute of Research, 503 Robert Grant Avenue, Silver Spring, MD 20910 USA; 30 Department of Internal Medicine, Division of Intensive Care and Emergency Medicine, Medical University of Innsbruck, Anichstr. 35, 6020 Innsbruck, Austria; 31 Department of Internal Medicine I, Division of Infectious Diseases, Medical University of Vienna, Währinger Gürtel 18 – 20, 1090 Vienna, Austria; 32 Department of Respiratory Medicine, Hannover Medical School, Carl-Neuberg-Strasse 1, 30625 Hannover, Germany; 33 Assign Data Management and Biostatistics GmbH, Stadlweg 23, 6020 Innsbruck, Austria; 34 Valneva Austria GmbH, Campus Vienna Biocenter 3, 1030 Vienna, Austria; 35 Universitat Autonoma de Barcelona, Barcelona, Spain

**Keywords:** Pseudomonas aeruginosa, Vaccination, Immunity, Immunocompromised host, Bacterial infections, Mortality

## Abstract

**Background:**

Currently, no vaccine against *Pseudomonas* is available. IC43 is a new, recombinant, protein (OprF/I)-based vaccine against the opportunistic pathogen, *Pseudomonas aeruginosa*, a major cause of serious hospital-acquired infections. IC43 has proven immunogenicity and tolerability in healthy volunteers, patients with burns, and patients with chronic lung diseases. In order to assess the immunogenicity and safety of IC43 in patients who are most at risk of acquiring *Pseudomonas* infections, it was evaluated in mechanically ventilated ICU patients.

**Methods:**

We conducted a randomized, placebo-controlled, partially blinded study in mechanically ventilated ICU patients. The immunogenicity of IC43 at day 14 was determined as the primary endpoint, and safety, efficacy against *P. aeruginosa* infections, and all-cause mortality were evaluated as secondary endpoints. Vaccinations (100 μg or 200 μg IC43 with adjuvant, or 100 μg IC43 without adjuvant, or placebo) were given twice in a 7-day interval and patients were followed up for 90 days.

**Results:**

Higher OprF/I IgG antibody titers were seen at day 14 for all IC43 groups versus placebo (*P* < 0.0001). Seroconversion (≥4-fold increase in OprF/I IgG titer from days 0 to 14) was highest with 100 μg IC43 without adjuvant (80.6%). There were no significant differences in *P. aeruginosa* infection rates, with a low rate of invasive infections (pneumonia or bacteremia) in the IC43 groups (11.2-14.0%). Serious adverse events (SAEs) considered possibly related to therapy were reported by 2 patients (1.9%) in the group of 100 µg IC43 with adjuvant. Both SAEs resolved and no deaths were related to study treatment. Local tolerability symptoms were mild and rare (<5% of patients), a low rate of treatment-related treatment-emergent adverse events (3.1–10.6%) was observed in the IC43 groups.

**Conclusion:**

This phase II study has shown that IC43 vaccination of ventilated ICU patients produced a significant immunogenic effect. *P. aeruginosa* infection rates did not differ significantly between groups. In the absence of any difference in immune response following administration of 100 μg IC43 without adjuvant compared with 200 μg IC43 with adjuvant, the 100 μg dose without adjuvant was considered for further testing of its possible benefit of improved outcomes. There were no safety or mortality concerns.

**Trial registration:**

ClinicalTrials.gov, NCT00876252. Registered on 3 April 2009.

**Electronic supplementary material:**

The online version of this article (doi:10.1186/s13054-017-1601-9) contains supplementary material, which is available to authorized users.

## Background

The opportunistic bacterial pathogen *Pseudomonas aeruginosa* is a major cause of serious hospital-acquired infections [1]. *P. aeruginosa* is a particular problem for seriously ill patients in intensive care units (ICUs), with important associated infections being ventilator-associated pneumonia, catheter-related bloodstream infections, and catheter-associated urinary tract infections [2–4]. The risk of *Pseudomonas* infections increases with duration of ICU stay, and infection is associated with an increased risk of mortality [5].

Effective treatment of *P. aeruginosa* infection is hindered by the organism’s ability to develop resistance to antibacterial agents, even during the course of treatment against the infection [2, 6]. The escalating prevalence of antibiotic resistance in *P. aeruginosa* requires development of new strategies. Vaccine research has included approaches to generating antibodies to surface molecules, such as lipopolysaccharide and outer membrane proteins [7], and a role for T-helper type 17 (Th17)-stimulating protein antigens has been proposed [8]. However, there is currently no vaccine available for *P. aeruginosa*.

Vaccines that are based on outer membrane proteins are attractive candidates because outer membrane proteins are conserved among all the 17 known serotypes of *P. aeruginosa*, and are still present after phenotypic conversion of *P. aeruginosa* [9]. IC43 (Valneva, Vienna, Austria) is a recombinant outer membrane protein (Opr)-based vaccine against *P. aeruginosa*, consisting of epitopes of OprF and OprI. It has shown promising results in healthy volunteers, patients with burns, and patients with chronic lung disease [10–15], with proven immunogenicity at individual doses of 500–1000 μg via intranasal application, and 20–500 μg via intramuscular injection. Confirmation of the optimal dose in the target population of patients at particularly high risk of acquiring *P. aeruginosa* infections, such as ICU patients, is now required.

We present herein the results of a dose-finding study of IC43 in ventilated ICU patients. The primary objective was to assess the immunogenicity of IC43 at doses of 100 μg and 200 μg with adjuvant, respectively, or 100 μg without adjuvant, 14 days after the first vaccination. Secondary objectives were to investigate immunogenicity up to day 90, safety and tolerability, to estimate the rate of *P. aeruginosa* infections, and to analyze the impact of IC43 vaccination on other factors, including overall survival. None of the results of this study have been previously reported.

## Methods

Additional detail on the methods is provided in Additional file 1.

### Trial design

**Fig. 1:**
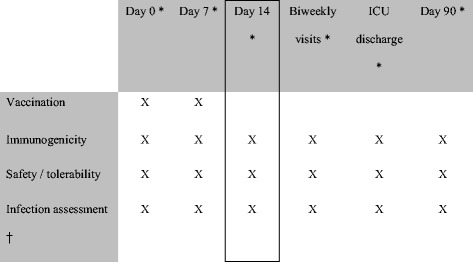
Study design. *Day 0 assessments were in the intensive care unit (*ICU*). Subsequent visits were performed in the ICU, hospital, or outpatient setting. Key study visits were at days 0, 7, 14, and 90, and ICU discharge. Optional study visits were performed on days 28, 42, 56, and 70, and only if the patient was still in the ICU or hospital. The total number of visits was dependent on the length of hospital stay (maximum 9 visits). The day-90 visit was considered essential; if patients were not able to attend in person, a telephone call for safety assessment was conducted. The primary endpoint (immunogenicity assessment) was outer membrane protein (OprF/I)-specific immunoglobulin G (IgG) antibody titer at day 14. †Surveillance cultures for *Pseudomonas aeruginosa* (*P. aeruginosa*) diagnosis were taken from blood, wounds (if applicable), respiratory tract, urine, and central venous catheter at visits conducted in the ICU. In between these visits, and at other visits up to day 90, cultures for *P. aeruginosa* diagnosis were taken at the investigator’s discretion, if medically indicated

This was a phase II, randomized, placebo-controlled, partially blinded, parallel-group, multicenter study to assess the immunogenicity and safety of IC43 vaccination in mechanically ventilated ICU patients. Doses of 100 μg and 200 μg with adjuvant and a dose of 100 μg without adjuvant were tested. Patients were enrolled, randomized and vaccinated on day 0. A second vaccination was given on day 7. Clinical study visits were performed up to day 90 (Fig. 1).

Subjects were initially randomized in a 1-1-1 ratio to IC43 100 μg, IC43 200 μg, or placebo (with both IC43 doses given with aluminum hydroxide adjuvant). After 137 patients had been enrolled, data became available from a phase-I trial, in which both 100 μg IC43 without adjuvant and 100 μg IC43 with adjuvant had a favorable safety profile and a similar immunogenicity profile. Based on the newly available data and following the recommendation of a Data Safety Monitoring Board (DSMB), a protocol amendment was introduced to add a fourth treatment group in this phase II study, to assess IC43 100 μg without aluminum hydroxide adjuvant. From this point in the study, randomization was performed in a 2:1:1:1 ratio (double the number of patients randomized to 100 μg IC43 without adjuvant to achieve equal numbers per group).

The randomization lists were generated by a statistician at a contract research organization (Assign Data Management and Biostatistics GmbH, Innsbruck, Austria), using nQuery Advisor. A hard copy and the electronic files were stored in a bank deposit until the study was un-blinded. The first randomization code had a block size of 3 (1 × IC43 100 μg, 1 × IC43 200 μg, and 1 × placebo). After the protocol amendment and introduction of the fourth treatment group, the code had a block size of 5 (1 × IC43 100 μg, 1 × IC43 200 μg, 2 × IC43 100 μg without adjuvant, and 1 × placebo). The randomization was stratified by study center. Each subject was assigned the next free medication kit (randomization) number for the corresponding study center in ascending order.

A blinded independent Clinical Endpoint Committee (CEC) reviewed and confirmed diagnosis of *P. aeruginosa* infections. A blinded independent DSMB evaluated safety data and provided recommendations to the sponsor.

### Study population

The main inclusion criteria were: male or females, 18–80 years of age; ICU patients on mechanical ventilation with an expected need of continued mechanical ventilation for >48 hours after inclusion (study inclusion had to occur as soon as possible after start of mechanical ventilation); a high probability of survival for ≥48 hours; and written informed consent (e.g. by the patient or legal representative) or waiver according to national regulations.

Patients were excluded if they had low severity of illness (i.e. acute physiology score <8 at visit 0; 12 physiological values, clinically worst value was recorded, higher scores indicate more severe illness), were <6 months post-organ transplantation, had severe thrombocytopenia or other coagulopathy making them unsuitable for intramuscular injection, had expected plasmapheresis or immune-adsorption during the study period, had used any other investigational or non-registered drug (except the study vaccine) within 30 days prior to IC43 vaccination at day 0, were pregnant or lactating, or had been committed involuntarily to an institution.

The study was performed at 34 sites, mainly public hospitals or university hospitals, in Europe (Austria, Romania, Belgium, Hungary, Spain, and Turkey) and South America (Argentina and Chile).

### Study vaccine

The IC43 vaccine (Met-Ala-(His)_6_-OprF_190–342_-OprI_21–83_) consists of the C-terminal part of OprF and the entire outer membrane protein OprI with six histidine residues fused to the N-terminus of the recombinant protein (OprF/I). IC43 was produced in *Escherichia coli*. The purified protein was stored in phosphate-buffered saline (PBS) solution and diluted in 0.9% sodium chloride. The protein was absorbed to aluminum hydroxide as adjuvant (400 μg protein per ml aluminum hydroxide) or directly filled in glass vials. The final protein concentration was 100 μg/ml. The placebo was PBS solution containing 0.9% sodium chloride and 400 μg aluminum hydroxide.

The drug substance (purified OprF/I fusion protein) was manufactured by Eurogentec S.A. Biologics, Liege Science Park-4102, Seraing, Belgium. The IC43 drug product and placebo for injection were produced by SynCo Biopartners B.V., Paasheuvelweg 30, 1105BJ Amsterdam ZO, The Netherlands. All study drugs were produced and released in accordance with Good Manufacturing Practice.

IC43 with adjuvant and placebo were stored in refrigerators at 2–8 °C. IC43 without adjuvant was delivered on dry ice and stored in freezers at −20 °C (+/− 5 °C). IC43 with adjuvant and placebo vials were shaken before use. Vials containing IC43 without adjuvant were thawed for 40 minutes at room temperature, reversed 10 times before use, and administered within 30 minutes.

IC43 without adjuvant (i.e. purified OprF/I fusion protein) was administered un-blinded, and IC43 with adjuvant and placebo were administered blinded because of different appearance and storage conditions. Intramuscular injections were given in the deltoid region of the upper arm. Sealed envelopes containing details of treatment allocation (one per patient) were provided to study sites in case emergency un-blinding was required.

### Outcomes

The primary endpoint was immunogenicity at day 14, determined by OprF/I-specific immunoglobulin G (IgG) antibody titer. Subgroup analyses were performed for immunosuppression, gender and age. Secondary endpoints included OprF/I-specific IgG antibody titers on days 7, 28, 42, 56, 70, and 90, CEC-confirmed *P. aeruginosa* infections up to day 90, and overall survival, safety, and tolerability.

### Immunogenicity, efficacy, safety, and tolerability measures

Serum was collected for immunogenicity assessments (OprF/I ELISA) on days 0, 7, 14, 28, 42, 56, 70, 90, and ICU discharge. *P. aeruginosa* surveillance cultures were taken at each ICU study visit. Bacteremia was assessed by bacterial blood culture. Data from respiratory cultures and urine measurements were analyzed quantitatively and data from wounds were analyzed qualitatively. Semi-quantitative microbiological culture of central venous catheter samples was performed if possible, otherwise qualitative analysis was performed. In addition, cultures were collected at other times (i.e. between visits), at the investigator’s discretion, during the ICU and hospital stay.

The sequential organ failure assessment (SOFA) score (range 0–24, with scores for each organ system (respiratory, coagulation, liver, cardiovascular, central nervous system, and kidney) ranging from 0 (normal) to 4 (most abnormal)) was recorded at each visit during the ICU stay.

Adverse events (AEs) and serious adverse events (SAEs), clinical laboratory tests (hematology, clinical chemistry and urinalysis), and local tolerability and systemic tolerability assessments were performed at each visit. Local tolerability (i.e. reactions at the injection site) was evaluated by the investigator for up to 1 hour after each vaccination. At each visit, the injection site was also inspected and evaluated by the investigator. Grading was performed according to Food and Drug Administration Guidance for Industry [16], modified to reflect the guidance of the Brighton Collaboration. For each type of local reaction (erythema/redness, induration, pain, swelling, itching, or tenderness), a record was made of whether it was present and, if present the severity was graded as <1, mild (grade 1), moderate (grade 2), severe (grade 3) or potentially life threatening (grade 4).

For visits performed at the ICU, systemic tolerability of the vaccination was assessed by monitoring vital signs (pulse and blood pressure). Vital signs were monitored over a period of 1 hour after vaccination. If the second vaccination was performed at the hospital ward or in the outpatient setting, patients were observed by the investigator for 1 hour after vaccination for the assessment of local and systemic tolerability and for immediate treatment of possible side effects.

Adverse events were coded according to the Medical Dictionary for Regulatory Activities (MedDRA) coding dictionary version 11.1. Treatment-emergent adverse events (TEAEs) were defined as those events for which the first onset or worsening was simultaneous with or after the first vaccination.

### Sample size

A total of 400 patients (100/group) was required to provide power of 80% for the detection of a pairwise difference between the study groups in immunogenicity (primary study endpoint, day 14) (*t* test for independent comparisons), based on OprF/I-specific antibody titer data from a phase I study.

The sample size allowed for the estimation of an expected *P. aeruginosa* infection rate of 7% with a precision of +/− 5.0% (two-sided 95% confidence interval (CI)). This study was not powered to detect differences in *P. aeruginosa* infections between treatment groups. The intention for collecting *P. aeruginosa* infections was to investigate the epidemiology to prepare for an adequately powered phase III study.

### Statistical methods

Statistical analyses were performed using SAS® version 9.2 (SAS Institute, Cary, NC, USA). Primary immunogenicity analysis compared the OprF/I-specific IgG antibody geometric mean titer (GMT) on day 14 among the four treatment groups using the intention-to-treat (ITT) population. GMTs and GMT ratios were estimated by applying analysis of variance including pooled site and group as factors, using log 10-transformed data and taking the anti-log of the resulting point estimates for the least squares means, pair-wise least squares means differences and the corresponding 95% confidence intervals (CIs). Tukey’s honestly significant difference test was applied for pairwise comparisons. Similar analysis of OprF/I IgG titers was performed for other time points in the study (secondary analysis) and in subgroups based on immunosuppressive status, gender, and age.

### Seroconversion, *P. aeruginosa* infections, mortality, and SOFA score analyses

The number and percentage of patients with seroconversion (at least fourfold increase in OprF/I-specific IgG antibody titers from days 0 to 14) were summarized with 95% CIs calculated according to Wilson’s method, as recommended by Altman [17]. The number and percentage of patients with CEC-confirmed *P. aeruginosa* infections by event type was calculated (overall and by time of onset: baseline (day 0), up to day 7, between days 7 and 14, and between days 14 and 90). The Fisher-Freeman-Halton test was used to test for a difference among the treatment groups for each event type.

A Kaplan-Meier survival curve was calculated by treatment group to show time until death from the date of first vaccination (day 0). The post-hoc log-rank test and Cox regression analysis (with group as a factor) was used to compare mortality rates between the treatment groups at day 28. Additional post-hoc Cox regression analysis (with group as a factor) was applied to analyze time until death. Post-hoc Cox regression analysis was used to examine correlation between the OprF/I-specific IgG antibody titer and the overall mortality rate, using log-transformed values. Descriptive statistics were provided for the SOFA score.

### Safety and tolerability analyses

Descriptive statistics were used to summarize safety and tolerability data. For TEAEs, the Fisher-Freeman-Halton test was used to test for a difference among the treatment groups.

### Analysis populations

The ITT analysis population was defined as all randomized patients who received at least one vaccination. Patients were analyzed according to the treatment group to which they were randomized, rather than by the actual treatment received. Primary immunogenicity and efficacy analyses were based on the ITT population.

The safety population was defined as all patients who received at least one vaccination using the actual treatment received. The safety population was used for safety and tolerability analyses, demographic, and baseline data. The safety population and ITT population were identical.

The per-protocol (PP) population was defined as all randomized patients who received both vaccinations, excluding those subjects with at least one major protocol deviation. PP population analysis was considered supportive.

## Results

### Study participation

**Fig. 2:**
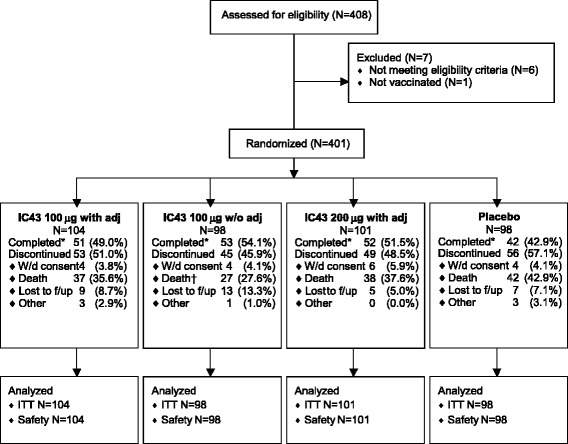
Patient disposition. *Dosage deviations were reported in 9 (2.5%), 11 (3.0%), 12 (3.2%), and 16 (4.5%) patients, in the IC43 100 μg with adjuvant, 100 μg without adjuvant, 200 μg with adjuvant, and placebo groups, respectively. Treatment assignment deviations were reported in 2 (0.6%), 5 (1.4%), 3 (0.8%), and 2 (0.6%) patients, respectively. †Early study terminations due to patient deaths, as documented in the case report form. In addition, a further patient from the group randomized to IC43 100 μg without adjuvant who died, and for whom the date and primary cause of death was missing, is not included in this figure. *f/up* follow up, *ITT* intention-to-treat, *W/d* withdrawn, *w/o adj* without aluminum hydroxide adjuvant, *with adj* with aluminum hydroxide adjuvant

The study was conducted between January 2009 and February 2010. Patient disposition is illustrated in Fig. 2. The groups were similar in terms of demographic and baseline characteristics (Table 1), reasons for ICU admission (Table 2), and the duration of ventilation, and ICU and hospital stay (Table 3). There were no major differences in the use of concomitant immunosuppressive agents, non-steroidal anti-inflammatory drugs, or anti-infective agents. In particular, there were no differences between the treatment groups in the numbers of patients receiving *P. aeruginosa*-relevant antibiotics (Table 4).

**Table 1:** Baseline demographic and clinical characteristics (ITT population)

Parameter^a^	IC43 100 μg with adj *N* = 104	IC43 100 μg w/o adj *N* = 98	IC43 200 μg with adj *N* = 101	Placebo *N* = 98	Total *N* = 401
Age (years)	57.0 (16.2)	54.3 (15.3)	55.2 (15.7)	58.1 (15.3)	56.1 (15.6)
Weight (kg)	82.0 (19.2)	84.6 (17.6)	82.5 (17.9)	82.5 (21.7)	82.9 (19.1)
Height (cm)	170.8 (10.5)	173.5 (9.1)	171.5 (8.2)	171.5 (10.0)	171.8 (9.5)
Sex					
Male	64 (61.5)	70 (71.4)	72 (71.3)	61 (62.2)	267 (66.6)
Female	40 (38.5)	28 (28.6)	29 (28.7)	37 (37.8)	134 (33.4)
Race					
Caucasian	95 (91.3)	93 (94.9)	94 93.1)	92 (93.9)	374 (93.3)
Asian	2 (1.9)	1 (1.0)	0 (0.0)	2 (2.0)	5 (1.2)
Black	1 (1.0)	0 (0.0)	2 (2.0)	1 (1.0)	4 (1.0)
Other	6 (5.8)	4 (4.1)	5 (5.0)	3 (3.1)	18 (4.5)
HIV status					
Positive	0 (0.0)	2 (2.0)	0 (0.0)	0 (0.0)	2 (0.5)
Missing	2 (1.9)	1 (1.0)	3 (3.0)	3 (3.1)	9 (2.2)

^a^Data are presented as mean (standard deviation) for age, weight and height, and number (%) of patients for sex, race, and HIV status. *HIV* human immunodeficiency virus, *ITT* intention-to-treat, *w/o adj* without aluminum hydroxide adjuvant, *with adj* with aluminum hydroxide adjuvant

**Table 2:** Reasons for first admission to intensive care unit (ITT population)

Reason for first admission^a^	IC43 100 μg with adj (*N* = 104) Number (%)	IC43 100 μg w/o adj (*N* = 98) Number (%)	IC43 200 μg with adj (*N* = 101) Number (%)	Placebo (*N* = 98) Number (%)	Total (*N* = 401) Number (%)
Medical^b^	82 (78.8)	75 (76.5)	80 (79.2)	74 (75.5)	311 (77.6)
Surgical	8 (7.7)	4 (4.1)	9 (8.9)	11 (11.2)	32 (8.0)
Traumatic	14 (13.5)	19 (19.4)	12 (11.9)	12 (12.2)	57 (14.2)
Unclassifiable	0 (0.0)	0 (0.0)	0 (0.0)	1 (1.0)	1 (0.2)

^a^Patients may have been admitted more than once; only the first admission is classified. ^b^Includes cardiopulmonary resuscitation, stroke, sepsis, respiratory insufficiency, and cardiac arrest. *ITT* intention-to-treat; *w/o adj* without aluminum hydroxide adjuvant, *with adj* with aluminum hydroxide adjuvant

**Table 3:** Duration of ventilation, intensive care unit stay, and hospital stay (safety population)

Duration (days)^a^	IC43 100 μg with adj (*N* = 104)	IC43 100 μg w/o adj (*N* = 98)	IC43 200 μg with adj (*N* = 101)	Placebo (*N* = 98)	Total (*N* = 401)
Ventilation	18.5 (13.4)	18.7 (18.6)	18.5 (17.2)	17.9 (14.8)	18.4 (16.0)
ICU stay	26.6 (17.7)	24.3 (17.8)	24.0 (16.9)	23.5 (16.0)	24.6 (17.1)
Hospital stay^b^	15.2 (20.3)	13.9 (14.6)	14.4 (20.9)	12.4 (17.6)	14.0 (18.5)

^a^Data are presented as mean (standard deviation); includes non-censored data only. Data were either censored or missing for 14 patients (ventilation), 11 patients (ICU stay) and 32 patients (hospital stay). ^b^Not including ICU stay. *ICU* intensive care unit, *w/o adj* without aluminum hydroxide adjuvant, *with adj* with aluminum hydroxide adjuvant

**Table 4:** Number of patients receiving *P. aeruginosa* relevant antibiotics ﻿﻿(﻿ITT population)

	IC43 100 µg with adj(*N* = 104) No. (%)	IC43 100 µg w/o adj(*N* = 98) No. (%)	IC43 200 µg with adj(*N* = 101) No. (%)	Placebo(*N* = 98) No. (%)	Total(*N* = 401) No. (%)
Subjects with at least one *P. aeruginosa* relevant antibiotics during the study	84 (80.8)	76 (77.6)	84 (83.2)	83 (84.7)	327 (81.5)

*ITT* intention-to-treat; *w/o adj* without aluminium hydroxide adjuvant; *with adj* with aluminium hydroxide adjuvant

### Immunogenicity data

**Fig. 3:**
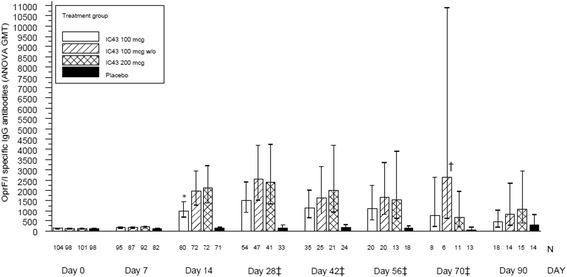
Outer membrane protein F/I hybrid vaccine (*OprF/I*)-specific IgG antibody geometric mean titers (*GMT*) (U/ml) (intention-to-treat (*ITT*) population). Note, on day 14 (primary endpoint), there was a statistically significant difference in the OprF/I-specific IgG antibody titer in all IC43 groups compared with placebo (all *P* < 0.0001). In addition, there were statistically significant differences between all IC43 groups versus placebo on days 28, 42, 56, and 70 (*P* ≤ 0.0119). At baseline (day 0), 25 subjects had a detectable OprF/I IgG titer >350 U/ml; none of these patients had a baseline infection. *Statistically significant difference between 200 μg and 100 μg IC43 with adjuvant (*P* = 0.0344). ^†^Two outliers representing measurements in six subjects (11,582 and 7,935 U/ml). ^‡^Optional visit. *w/o* without aluminum hydroxide adjuvant, *ANOVA* analysis of variance

OprF/I-specific IgG antibody titers are illustrated for each study visit in Fig. 3. For the primary endpoint, OprF/I-specific IgG antibody titer on day 14, there was a statistically significant overall treatment effect (*P* < 0.0001; ITT population). On pairwise comparisons there were statistically significantly higher titers in each of the three IC43 treatment groups on day 14, compared with placebo (all *P* < 0.0001), thus demonstrating the presence of an immune response following vaccination with both IC43 doses and formulations. There was no difference in immune response on day 14 following the 100 μg dose with or without adjuvant (*P* = 0.0735). However, there was a statistically significant difference between the doses of 100 μg and 200 μg with adjuvant (*P* = 0.0344), with a greater immune response to the higher dose, indicating a dose-response relationship in the groups randomized to IC43 with adjuvant. However, on day 14 the 200 μg dose with adjuvant was not more immunogenic than the 100 μg dose without adjuvant (*P* = 0.9933).

In the subgroup analyses of OprF/I-specific IgG antibody titer on day 14 by immunosuppressive status, a statistically significant overall treatment effect was detected in patients with high and with low immunosuppression (*P* = 0.0001 and *P* = 0.0429, respectively), showing that the immune response was present irrespective of immunosuppressive status. Similarly, significant treatment effects were noted on day 14 in both gender subgroups (i.e. male and female patients) and both age subgroups (<65 and ≥65 years) (all *P* < 0.0001), indicating that the immune response following IC43 vaccination was present in both genders and age groups. Pairwise comparisons showed the OprF/I-specific IgG antibody titer on day 14 was significantly higher in the group randomized to 200 μg with adjuvant compared with group randomized to 100 μg with adjuvant among male patients and in patients aged <65 years (*P* = 0.0319 and *P* = 0.0142, respectively), reflecting the dose-response relationship for the groups receiving IC43 with adjuvant, which was observed in the ITT population. However, the 200 μg dose with adjuvant was not more immunogenic than the 100 μg dose without adjuvant in these subgroups, also reflecting the overall ITT population.

Seroconversion (≥4-fold increase in OprF/I-specific IgG antibody titer from days 0 to 14) was observed in ≥65.0% of patients in the IC43 treatment groups and 7.0% of patients in the placebo group (Table 5). Seroconversion was highest in the group receiving 100 μg IC43 without adjuvant (80.6%).

**Table 5:** Seroconversion rates from days 0 to 14 (ITT population)

Treatment group	Patients seroconverted by day 14^a^ *n* (*N*)	Percentage	95% Confidence interval^b^
IC43 100 μg with adj	52 (80)	65.0%	(54.1%, 74.5%)
IC43 100 μg w/o adj	58 (72)	80.6%	(70.0%, 88.0%)
IC43 200 μg with adj	54 (72)	75.0%	(63.9%, 83.6%)
Placebo	5 (71)	7.0%	(3.0%, 15.4%)

^a^Seroconversion defined as ≥4-fold increase in Outer membrane protein F/I hybrid vaccine (OprF/I)-specific IgG antibody titer from days 0 to 14. ^b^95% Confidence interval calculated according to Wilson’s method, as recommended by Altman [17]. *﻿ITT* int﻿ention-to-treat﻿, *n* number of patients seroconverted, *N* number of patients with data, *w/o adj* without aluminum hydroxide adjuvant, *with adj* with aluminum hydroxide adjuvant

### *P. aeruginosa* infection rates

Data from patients with any *P. aeruginosa*-positive culture result were evaluated by the CEC. In total, results from 148 patients were reviewed by the CEC, with a similar percentage of patients in each treatment group: 39 (37.5%), 37 (37.8%), 40 (39.6%), and 32 (32.7%) patients in the groups randomized to 100 μg with adjuvant, 100 μg without adjuvant, 200 μg with adjuvant, or placebo, respectively (ITT population).

Invasive infections (pneumonia or bacteremia) were reported by 11.2–14.0% of patients in the IC43 groups and 6.1% subjects in the placebo group (Table 6). Differences in the *P. aeruginosa* infection types were not statistically significantly different between treatment groups; hence, no conclusion can be drawn on a treatment effect.

**Table 6:** Confirmed *P. aeruginosa* infections up to day 90 (overall and stratified by time period for first time of onset) (ITT population)

Culture type^a^	Time period	IC43 100 μg with adj		IC43 100 μg w/o adj		IC43 200 μg with adj		Placebo		Overall
		*N* = 104		*N* = 98		*N* = 101		*N* = 98		*P* value^b^
		*n* (%)	*N*’	*n* (%)	*N*’	n (%)	*N*’	*n* (%)	*N*’	
Invasive	Overall	14 (13.5)	104	11 (11.2)	98	14 (14.0)	100	6 (6.1)	98	0.2533
infection^c^	Day 0 (baseline)	3 (2.9)	104	0 (0.0)	98	4 (4.0)	100	0 (0.0)	98	
	Days 0 to ≤7^d^	7 (6.7)	104	5 (5.1)	98	9 (9.0)	100	4 (4.1)	98	
	>Days 7 to ≤14	2 (2.1)	97	2 (2.2)	89	4 (4.4)	90	1 (1.3)	79	
	>Days 14 ≤ 90	5 (6.0)	84	4 (4.9)	81	1 (1.3)	79	1 (1.4)	74	
Bacteremia	Overall	10 (9.6)	104	7 (7.1)	98	9 (8.9)	101	6 (6.1)	98	0.7943
	Day 0 (baseline)	1 (1.0)	104	0 (0.0)	98	1 (1.0)	101	0 (0.0)	98	
	Days 0 to ≤7^d^	4 (3.8)	104	5 (5.1)	98	5 (5.0)	101	4 (4.1)	98	
	>Days 7 to ≤14	1 (1.0)	97	1 (1.1)	89	3 (3.3)	91	1 (1.3)	79	
	>Days 14 ≤ 90	5 (6.0)	84	1 (1.2)	81	1 (1.3)	80	1 (1.4)	74	
Pneumonia	Overall	5 (4.8)	104	4 (4.1)	98	6 (6.0)	100	0 (0.0)	98	0.0693
	Day 0 (baseline)	2 (2.0)	104	0 (0.0)	98	4 (4.0)	100	0 (0.0)	98	
	Days 0 to ≤7^d^	4 (3.8)	104	0 (0.0)	98	5 (5.0)	100	0 (0.0)	98	
	>Days 7 to ≤14	1 (1.0)	97	1 (1.1)	89	1 (1.1)	90	0 (0.0)	79	
	>Days 14 ≤ 90	0 (0.0)	84	3 (3.7)	81	0 (0.0)	79	0 (0.0)	74	
Tracheo-bronchitis	Overall	5 (4.8)	104	12 (12.2)	98	9 (9.0)	100	8 (8.2)	98	0.3022
	Day 0 (baseline)	0 (0.0)	104	1 (1.0)	98	1 (1.0)	100	0 (0.0)	98	
	Days 0 to ≤7^d^	3 (2.9)	104	4 (4.1)	98	4 (4.0)	100	5 (5.1)	98	
	>Days 7 to ≤14	1 (1.0)	97	6 (6.7)	89	0 (0.0)	90	0 (0.0)	79	
	>Days 14 ≤ 90	1 (1.2)	84	2 (2.5)	81	5 (6.3)	79	2 (2.7)	74	
Central venous catheter infection	Overall	1 (1.2)	84	0 (0.0)	74	3 (4.1)	73	2 (2.6)	76	0.2863
	Day 0 (baseline)	0 (0.0)	84	0 (0.0)	74	0 (0.0)	73	0 (0.0)	76	
	Days 0 to ≤7^d^	0 (0.0)	84	0 (0.0)	74	1 (1.4)	73	0 (0.0)	76	
	>Days 7 to ≤14	0 (0.0)	77	0 (0.0)	67	0 (0.0)	66	0 (0.0)	60	
	>Days 14 ≤ 90	1 (1.6)	64	0 (0.0)	61	2 (3.5)	57	2 (3.6)	55	
Wound infection	Overall	9 (11.0)	82	6 (8.5)	71	8 (10.7)	75	6 (7.8)	77	0.8960
	Day 0 (baseline)	1 (1.2)	82	0 (0.0)	71	2 (2.7)	75	1 (1.3)	77	
	Days 0 to ≤7^d^	4 (4.9)	82	2 (2.8)	71	3 (4.0)	75	2 (2.6)	77	
	>Days 7 to ≤14	0 (0.0)	75	2 (3.1)	64	1 (1.5)	67	2 (3.3)	61	
	>Days 14 ≤ 90	5 (8.1)	62	2 (3.4)	58	4 (6.9)	58	2 (3.5)	57	
Urinary tract infection	Overall	4 (3.8)	104	5 (5.1)	98	7 (6.9)	101	9 (9.3)	97	0.4380
	Day 0 (baseline)	0 (0.0)	104	0 (0.0)	98	1 (1.0)	101	0 (0.0)	97	
	Days 0 to ≤7^d^	0 (0.0)	104	1 (1.0)	98	2 (2.0)	101	1 (1.0)	97	
	>Days 7 to ≤14	2 (2.1)	97	0 (0.0)	89	2 (2.2)	91	3 (3.8)	78	
	>Days 14 ≤ 90	2 (2.4)	84	4 (4.9)	81	3 (3.8)	80	5 (6.8)	73	

Note, percentages are based on the numbers of non-missing observations (*n*). *ITT* intention-to-treat, *n* (%) number (%) of patients, *N* number of patients in treatment group, *N’* number of patients with assessable data at start of the observation period, *w/o adj* without aluminum hydroxide adjuvant, *with adj* with aluminum hydroxide adjuvant. ^a^Confirmed by Clinical Endpoint Committee. ^b^Fisher-Freeman-Halton test, ^c^bacteremia or pneumonia. ^d^Data from day 0 (baseline) are included in the row for days 0 to ≤7. Therefore, the data for *Overall* include the rows for days 0 to ≤7, >days 7 to ≤14, and > days 14 to ≤90 rows, but not row for day 0 (baseline)

### Mortality

**Fig. 4:**
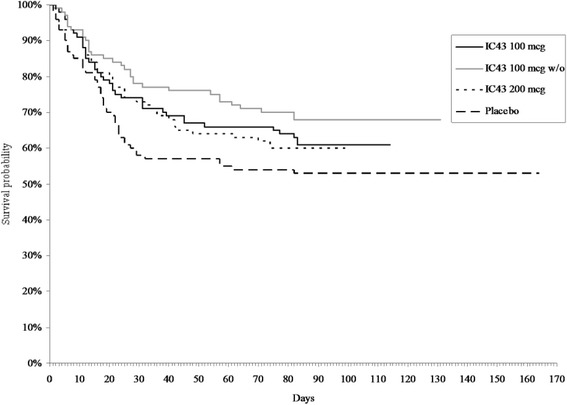
Survival curve: time until death by treatment group. In the group randomized to 100 μg IC43 without adjuvant, there was statistically significantly lower mortality versus placebo by day 28 (*P* = 0.0099, log-rank and Cox regression analysis) and significantly longer survival during the study versus placebo (*P* = 0.0196, Cox regression analysis). *w/o* without aluminum hydroxide adjuvant

Survival curves are shown in Fig. 4. By day 28, the number (percentage) of patients who had died was 26 (26.8%), 20 (21.7%), 25 (26.0%) and 36 (40.0%) in the groups randomized to 100 μg IC43 with adjuvant, 100 μg IC43 without adjuvant, 200 μg IC43 with adjuvant, and placebo, respectively (safety population). Corresponding data for overall (study end) mortality were 37 (35.6%), 28 (28.6%), 38 (37.6%), and 42 (42.9%) patients, respectively.

There were no major differences among the treatment groups in terms of mean (and median) SOFA score on day 0 (baseline). The median baseline SOFA score was highest in the placebo group (9.0 versus 8.0 in each of the IC43 groups) (Table 7). However, by day 14 the median SOFA score was lowest in the placebo group (4.0 versus 5.0, 5.5, and 6.0 with the IC43 100 μg dose with adjuvant, the IC43 100 μg dose without adjuvant, and the IC43 200 μg dose with adjuvant, respectively), even though mortality appeared to be higher in the placebo group than in the IC43 groups from day 14 onward (Fig. 4).

**Table 7:** SOFA score on days 0 (baseline), 7, and 14 (safety population)

SOFA score	Parameter	IC43 100 μg with adj (N = 104)	IC43 100 μg w/o adj (N = 98)	IC43 200 μg with adj (N = 101)	Placebo (N = 98)
Day 0	*n*	104	98	101	98
(baseline)	Mean (SD)	8.6 (3.5)	7.8 (3.5)	8.0 (3.2)	8.7 (3.7)
	Median	8.0	8.0	8.0	9.0
Day 7	*n*	88	79	78	79
	Mean (SD)	6.3 (4.1)	5.9 (4.1)	5.9 (3.6)	6.7 (4.5)
	Median	6.0	5.0	6.0	5.0
Day 14	*n*	61	48	49	50
	Mean (SD)	5.6 (3.8)	6.2 (4.1)	7.1 (4.5)	5.5 (4.7)
	Median	5.0	5.5	6.0	4.0

*n* number of patients, *SD* standard deviation, *SOFA* sequential organ failure assessment, *w/o adj* without aluminum hydroxide adjuvant, *with adj* with aluminum hydroxide adjuvant

Although there was some variance in the mortality per study center, a cox regression analysis showed that on a treatment level, the observed effect between the groups randomized to 100 μg IC43 without adjuvant or to placebo remained significant (*P* = 0.0079).

### Safety and tolerability

The proportion of patients who experienced at least one TEAE was similar across the treatment groups (95 (91.3%), 83 (84.7%), 87 (86.1%) and 87 (88.8%) patients in the groups randomized to 100 μg IC43 with adjuvant, 100 μg without adjuvant, 200 μg with adjuvant, or placebo, respectively (safety population)) with no statistically significant difference among groups (*P* > 0.05, Fisher-Freeman-Halton test). The percentage of patients who had at least one treatment-related TEAE was small (3.1 − 10.6% in the IC43 groups, and 6.1% in the placebo group), with no statistically significant difference among the groups (*P* > 0.05, Fisher-Freeman-Halton test). Overall, the number and nature of treatment-related TEAEs did not point to any safety concern.

SAEs were reported by 59 (56.7%), 38 (38.8%), 52 (51.5%), and 53 (54.1%) patients in the groups randomized to 100 μg IC43 with adjuvant, 100 μg without adjuvant, 200 μg with adjuvant, or placebo, respectively, with no statistically significant difference among the groups (*P* > 0.05, Fisher-Freeman-Halton test). None of the deaths was considered by the investigator to be related to study treatment. SAEs (pulmonary hemorrhage and shock) that were considered to be possibly related to treatment were reported by 2 (1.9%) patients in the group receiving 100 μg IC43 with adjuvant. These two events were also the only cases of related severe events. Both SAEs resolved. Local tolerability symptoms, such as erythema, pain, tenderness (if assessable in sedated or unconscious patients) were mild and rare (<5% of patients). Evaluation of laboratory parameters did not indicate any safety issues.

## Discussion

This phase II study investigated two doses and two formulations of a *P. aeruginosa* vaccine in ventilated ICU patients for immunogenicity as the primary study endpoint, and for safety and efficacy as secondary outcomes. Both doses of IC43 (100 μg and 200 μg) were effective at producing an immune response by day 14 (7 days after the second vaccination), as measured by OprF/I-specific IgG antibody titer, and the immunogenic effect persisted until day 70.

The IC43 vaccine entails a 6xHis-tag; however, in general there were no objections from the regulators against the use of the His-tagged protein. The vaccine has been applied in two clinical studies, including the present one and there were no safety concerns observed. Hence there is currently no indication that the 6xHis-tagged IC43 vaccine would induce any adverse events due to the His-tag.

The addition of aluminum hydroxide adjuvant to IC43 did not appear to improve immunogenicity. The observation that the antigen is as immunogenic without adjuvant as it is with adjuvant is surprising, but has been seen in a previous study with IC43 in healthy volunteers [18] and in other studies with similar recombinant protein subunit vaccines, such as a *Staphylococcus aureus* vaccine candidate [19]. The immune response was observed irrespective of immunosuppressive status, gender, and age. There was also some increase in OprF/I IgG in placebo recipients, which is most likely linked to natural exposure to *P. aeruginosa*. Safety parameters indicated a good safety profile in all IC43 groups and IC43 was well-tolerated up to doses of 200 μg.

This study found no difference in *P. aeruginosa* infection rates between patients vaccinated with IC43 and placebo. However, the onset of infections was generally early, often in the first week after admission to the ICU and likely before the induction of an immune response by vaccination. Over 70% of *P. aeruginosa* invasive infections occurred before day 14, whereas the OprF/I-specific IgG immune response only emerged between days 7 and 14. It is thus likely that infections with an early onset might not be preventable by the vaccine.

It should also be noted that the study was insufficiently powered for efficacy in terms of *P. aeruginosa* infection rates, and the study methodology is likely to have biased detection of infections up to and including the day-14 visit (e.g. surveillance samples to test for infection were taken at specified study time points during the ICU stay only, mainly on days 7 and 14, because the mean ICU stay was <28 days). It is possible that the IC43 vaccine affects *P. aeruginosa* virulence rather than clearance. This would not affect the infection rate, but it would confer a potential benefit to patients that would need to be measured by the use of additional endpoints.

Mortality was assessed as a secondary endpoint. Mortality in the group receiving 100 μg IC43 without adjuvant, was significantly lower than in the placebo group by day 28. Additionally, OprF/I-specific IgG antibody titer on day 14 was found to be a significant prognostic factor for overall survival. Notably, the study was not powered to detect a difference in mortality rates, and mortality should be explored further in future trials.

The mechanism of action of IC43 is currently unknown. Speculation on possible mechanisms centers on effects mediated by the immunologically active outer membrane proteins F and I expressed as antigens in the recombinant vaccine. Little is known about the function of OprI; however, the OprF protein is critical in the full virulence of *Pseudomonas* as it mediates stimulation of the quorum sensing network [20], which includes binding of human interferon-gamma [21]. It is not clear whether antibodies to these two proteins will inhibit or modify their amplifying actions, although previous studies showed that OprF/I vaccine-induced antibodies inhibits binding of *P. aeruginosa* to interferon-gamma, suggesting an alternative mechanism [22].

We are aware that there are several confounders for mortality rates reported in nosocomial infections, particularly in ICU studies. Indeed, there is strong correlation between mortality and high SOFA scores on ICU admission [23]. We did not notice major differences between the treatment groups in SOFA scores at baseline or by day 14. The median SOFA score was lowest in the placebo group, and further studies should be controlled by severity of illness because the effect on outcomes is reduced to intermediate ranges of severity. Thus, differences in SOFA score or severity of illness may have a different impact on the attributable mortality.

## Conclusions

In conclusion, our study has shown a significant immunogenic effect with IC43 vaccination in ventilated ICU patients. While it has been recognized that the development of a vaccine against *Pseudomonas* infection has not been successful for almost half a century [24], these data provide rationale that vaccination against nosocomial *Pseudomonas* infection may improve clinical outcome in mechanically ventilated patients exposed to nosocomial infections. In the absence of any difference in immune response following administration of 100 μg IC43 without adjuvant compared with 200 μg IC43 with adjuvant, the 100 μg dose without adjuvant was considered for further testing of its possible benefit of improved survival in a currently ongoing placebo-controlled phase II/III study.

## Key messages

- This phase II study has shown that IC43 vaccination produces a significant immunogenic effect in ICU patients on ventilation.
- *P. aeruginosa* infection rates did not differ significantly between groups.
- In the absence of any difference in immune response following administration of the 100 μg dose of IC43 without adjuvant compared with the 200 μg dose with adjuvant, the 100 μg dose without adjuvant was considered for further testing of its possible benefit of improved outcomes.
- There were no safety or mortality concerns.

## Additional files

Additional file 1: Additional details on methods. (DOC 62 kb)
Additional file 2: Ethics Committees. (DOCX 18 kb)

## Abbreviations

CEC: Clinical Endpoint Committee
DSMB: Data Safety Monitoring Board
GMT: Geometric mean titer
ICU: Intensive care unit
IgG: Immunoglobulin G
ITT: Intention-to-treat
Opr: Outer membrane protein
PP: Per protocol
SAE: Serious adverse event
SOFA: Sequential organ failure assessment
TEAE: Treatment-emergent adverse event
