# Supplementary material for: A randomized placebo-controlled phase II study of a Pseudomonas vaccine in ventilated ICU patients
Source: Crit Care. 2017 Feb 4;21:22. doi: 10.1186/s13054-017-1601-9 (PMC5291979; doi:10.1186/s13054-017-1601-9)
Supplement: Additional file 1: — Additional details on methods. (DOC 62 kb) [file 13054_2017_1601_MOESM1_ESM.doc]

**ONLINE ADDITIONAL DATA**

**A Randomized Placebo-controlled Phase II Study of a *Pseudomonas* Vaccine in Ventilated ICU Patients**

Jordi Rello, Claus-Georg Krenn, Gottfried J Locker, Ernst Pilger, Christian Madl, Laura Balica, Thierry Dugernier, Pierre-Francois Laterre, Herbert Spapen, Pieter Depuydt, Jean-Louis Vincent, Lajos Bogár, Zsuzsanna Szabó, Barbara Völgyes, Rafael Máñez, Nahit Cakar, Atilla Ramazanoglu, Arzu Topeli, Maria A Mastruzzo, Abel Jasovich, Christian G Remolif, Liliana del Carmen Soria, Max A Andresen Hernandez, Carolina Ruiz Balart, Ildikó Krémer, Zsolt Molnár, Frank von Sonnenburg, Arthur Lyons, Michael Joannidis, Heinz Burgmann, Tobias Welte, Anton Klingler, Romana Hochreiter, Kerstin Westritschnig

**Methods**

**Immunogenicity Assay**

Human serum obtained at each study visit was analyzed for OprF/I-specific IgG using a validated enzyme-linked immunosorbent assay (ELISA). Microtiter plates were coated with 1 µg/ml OprF/I, stored at 2°C to 8°C for 12 to 72 hours, washed with PBS - 0.05% Tween 20, and blocked using PBS - 0.05% Tween 20 with 2% bovine serum albumin (BSA, fraction V, Biomol Hamburg Germany). Eight serial 4-fold dilutions of each serum sample were applied in duplicate starting at 1:20 dilution. Control wells with no sample, a quality control sample and a reference standard, both prepared from sera generated during a phase 1 clinical trial (E11), were run on each plate. Plates were incubated at ambient temperature for 1 to 2 hours, washed, and rabbit anti-human IgG horseradish peroxidase conjugate (Dako, Vienna, Austria) diluted in PBS - 0.05% Tween 20 with 2% BSA was added for 1 to 2 hours. The presence of OprF/I-specific IgG immunoglobulins was detected by the addition of a substrate (Substrate solution I/II, Bender MedSystems, Vienna, Austria). Quantification of specific IgG was performed using the reference substance curve and four-parameter logistic fit and parallel line analysis using SoftMax Pro v5.2 Software (Molecular Devices, Sunnyvale, USA). Responses below the limit of quantitation of the ELISA (350 U/ml) were replaced with 100 U/ml.

**Diagnosis of *P. aeruginosa* infections**

The CEC reviewed data from all patients with a documented positive *P. aeruginosa* culture result and confirmed infection according to diagnosis criteria pre-defined in the study protocol. In the case of missing data, no confirmation of infection was done by the CEC.

**Diagnosis of *P. aeruginosa* bacteremia**

Diagnosis of bloodstream infection was defined as one positive blood culture for *P. aeruginosa* plus the presence of appropriate clinical symptoms.

**Diagnosis of *P. aeruginosa* pneumonia**

Diagnosis of *P. aeruginosa* pneumonia was made if the diagnosis criteria for ventilator-assisted pneumonia were fulfilled according to the National Nosocomial Infection Surveillance System (NNIS) (E22) and described by the Hospitals in Europe Link for Infection Control through Surveillance (HELICS) (E33):

Presence of a new or progressive lung infiltrate on chest radiography (or CT scan) plus at least two of the following:

- Core temperature >38°C or <36°C
- WBC count: >12 x10^9^/l or <4.0 x10^9^/l
- Purulent tracheobronchial secretion

**Diagnosis of *P. aeruginosa* tracheobronchitis**

Diagnosis of *P. aeruginosa* tracheobronchitis was made if the following diagnosis criteria were fulfilled:

Lack of lung infiltrate on chest radiography (or CT scan) plus at least two of the following:

- Core temperature >38°C or <36°C
- WBC count: >12 x10^9^/l or <4.0 x10^9^/l
- Purulent tracheobronchial secretion

**Diagnosis of central venous catheter infection**

Local central venous catheter infections were defined as one quantitative culture, obtained by Maki’s roll technique, ≥10^3^ colony-forming unit (CFU)/ml or semiquantitative culture >15 CFU, whereas general central venous catheter-related infection was defined as quantitative culture ≥10^3^ CFU/ml or semiquantitative culture >15 CFU plus clinical signs of sepsis.

**Diagnosis of wound infection**

Diagnosis of wound infection was based on organisms isolated from aseptically obtained culture or fluid or tissue from the wound.

**Diagnosis of urinary tract infection**

Urinary tract infection was defined as leucocyturia plus positive urine culture that was ≥10^5^ microorganisms per ml with no more than two species of microorganisms.

Leucocyturia was defined according to Centers for Disease Control and Prevention/NNIS definitions for nosocomial infections (2004) (E44), including a positive dipstick for leukocyte esterase and/or nitrate, and pyuria (defined as a urine specimen with >10 WBC/mm^3^ or >3 WBC/high power field of unspun urine).

**General points**

The use of the Day 0 OprF/I‑specific IgG antibody titer as a covariate in the statistical model for the primary immunogenicity analysis was considered, but it was omitted because baseline values were <350 U/ml (imputed 100 U/ml) for the majority of patients (approximately 94%) as determined from the interim analysis. The usage of this covariate was therefore deemed counterproductive.

A comparison with all IC43 groups combined (i.e., 100 μg with adjuvant, 100 μg without adjuvant, and 200 μg with adjuvant) versus placebo was planned to be performed only if there was no significant difference between the three IC43 treatment groups within the primary immunogenicity analysis (i.e., no significant overall treatment effect in the analysis of variance [ANOVA] with factors group and pooled site, between 100 μg with adjuvant, 100 μg without adjuvant and 200 μg with adjuvant, in the ITT population). These pooled analyses were not performed because the difference between the three IC43 treatment groups within the primary immunogenicity analysis was statistically significant (*P* = 0.032).

The factor ‘pooled sites’ was defined using the following standards: a site was not pooled with any other site if it had ≥5 patients within the per protocol (PP) population; a site was pooled with another site only if it had <5 patients within the PP population; pooling was only performed within one continent (sites in Turkey were considered European sites); and all sites for which pooling had to be performed within one continent were together in one pooled site.

Apart from the primary analysis, *P‑*values and CIs were interpreted in an exploratory manner. No adjustment for multiple testing was performed. The significance level was 0.05.

**Subgroup analysis**

In the subgroup analysis based on immunosuppressive status, a patient was considered ‘major’ immunosuppression if a positive human immunodeficiency virus (HIV) test was available or the patient had at least one intake of an immunosuppressive drug (identified by the Anatomical Therapeutic Chemical [ATC] code H02A, H02B or M01BA) in a ‘high’ dose between Day 0 and the day when the immunogenicity sample of Day 14 was taken (inclusive), as defined in the statistical analysis plan. Alternatively, patients were placed in the alternate group of ‘minor’ immunosuppressesion if a negative HIV test was available and they were either not receiving an immunosuppressive drug (identified by the ATC code H02A, H02B or M01BA) or were receiving an immunosuppressive drug in ‘low’ dose, between Day 0 and the day when the immunogenicity sample of Day 14 was taken (inclusive).

**Results**

Most infections were recorded on Days 0, 7 and 14, corresponding with the days when surveillance cultures were collected. XX were positive blood cultures. At other times, cultures were taken only if medically indicated (i.e., if the investigator suspected an infection), hence fewer infections were recorded at these times.

**References**

1. Mansouri E, Gabelsberger J, Knapp B, Hundt E, Lenz U, Hungerer KD, Gilleland HE, Jr, Staczek J, Domdey H, Von Specht BU. Safety and immunogenicity of a *Pseudomonas aeruginosa* hybrid outer membrane protein F-I vaccine in human volunteers. *Infect Immun* 1999;67(3):1461-1470.
2. Porzecanski I, Bowton DL. Diagnosis and treatment of ventilator-associated pneumonia. *Chest* 2006;130:597-604.
3. Suetens C, Morales I, Savey A, Palomar M, Hiesmayr M, Lepape A, Gastmeier P, Schmit JC, Valinteliene R, Fabry J. European surveillance of ICU-acquired infections (HELICS-ICU): methods and main results. *J Hosp Infect* 2007;65 Suppl 2:171-173.
4. Horan TC, Gaynes RP. Surveillance of nosocomial infections. Hospital Epidemiology and Infection Control, Mayhall CG. Philadelphia: Lippincott Williams & Wilkins, 2004:1659-1702.
