# Supplementary material for: A randomized placebo-controlled phase II study of a Pseudomonas vaccine in ventilated ICU patients
Source: Crit Care. 2017 Feb 4;21:22. doi: 10.1186/s13054-017-1601-9 (PMC5291979; doi:10.1186/s13054-017-1601-9)
Supplement: Additional file 2: — Ethics Committees. (DOCX 18 kb) [file 13054_2017_1601_MOESM2_ESM.docx]

**Ethics Committees**

**Austria** (Lead Ethics Committee):

Ethik-Kommission der Medizinischen Universität Wien

Borschkegasse 8b/E 06

1090 Wien

Reference number: 468/2008

**Belgium** (Lead Ethics Committee):

Université Catholique de Louvain

Faculté de Medicine

Commission d'Ethique Biomédicale Hospitalo-Facultaire de l'UCL

Avenue Hippocrates 55.14

1200 Bruxelles

Reference number: 2008/24OCT/296

**Hungary**:

Egeszegugyi Tudomanyos Tanacs

Klinika Farmakologiai Etikai Bizottsaga

Arany J. u. 6-8

1051 Budapest

Reference number: 6097-0/2009-1017EKL

**Romania**:

Comisia Nationala de Etica

Str. Aviator Sanatescu nr. 48, sect.1

011478 Bucuresti

No reference number provided

**Spain** (Lead Ethics Committee):

CEIC Hospital de Tarragona

Joan XXIII

Mallafre Guasch 4

43007 Tarragona

No reference number provided

**Turkey**:

I.Ü. Capa Tip Fakültesi Etik Kurulu

34390 Capa, Istanbul

No reference number provided

Marmara Universitesi Tip

Fakultesi Arastirma Etik Kurulu

Tophanelioglu Cad. 13/15

Altanuzade Uskudar, Istanbul

No reference number provided

Akdeniz Universitesi Tip

Fakultesi Etik Kurulu

Dumlupinar Bulvari/Kampus

07059 Antalya

No reference number provided

Hacettepe Üniversitesi Tıp Fakültesi Dekanlığı

Yerel Etik Kurul

06100 Sihhiye/Ankara

Reference number: 08/187

**Argentinia**:

Comité Indipendiente de Ética

Uriburu 774 Piso 1° C (C10227AAP)

Buenos Aires

No reference number provided

Comité de Docencia e

Investigación Hospital

Español de Mendoza

Dirección: Av. San Martín 965

CP 5501 Godoy Cruz

Provincia de Mendoza

No reference number provided

Comité de Docencia e Investigación

Hospital Zonal General de Agudos “Dr. Carlos Bocalandro”

Ruta 8

9100 Loma Hermosa

Partido 3 de Febrero

Provincia de Buenos Aires

No reference number provided

Comité de Docencia e Investigación

Fundación Sanatorio Guemes

Francisco Acuña de Figueroa 1240

(C1180AAV) Ciudad

Autónoma de Buenos Aires

No reference number provided

Comité de Docencia e Investigación Hospital Zonal

General de Agudos “Héroes de Malvinas”

Av. Ricardo Balbin 1910

Merlo (B1721FJN)

Provincia de Buenos Aires

No reference number provided

Comité de Docencia e Investigación

Hospital Central Mendoza

Alem y Salta (M5500GKO)

Mendoza Ciudad

Provincia de Mendoza

No reference number provided

**Chile**:

Comités de Evaluación Ético

Científico, Servicio de Salud

Metropolitano Sur-Oriente

Hospital Sotero del Rio

Ave. Concha y Toro 3459

Comuna de Puenta Alto

Santiago

No reference number provided
